# Advancing forest biodiversity visualisation through mixed reality

**DOI:** 10.1038/s41598-025-00285-y

**Published:** 2025-05-07

**Authors:** Cyprien R. Fol, Jiayan Zhao, Leonhard Späth, Arnadi Murtiyoso, Fabio Remondino, Verena C. Griess

**Affiliations:** 1 Forest Resources Management Group, ETH Zurich, Zurich, 8092 Switzerland; 2 Laboratory of Geo-Information Science and Remote Sensing, Wageningen University & Research, Wageningen, 6708 Netherlands; 3 Transdisciplinarity Lab, ETH Zurich, Zurich, 8092 Switzerland; 4 ICube Laboratory, INSA Strasbourg, Strasbourg, 67000 France; 5 3D Optical Metrology (3DOM) unit, Bruno Kessler Foundation (FBK), Trento, 38123 Italy

**Keywords:** Biodiversity inventory, 3D visualisation, Geometric accuracy, Heuristic evaluation, Information technology, Software, Forest ecology, Forestry

## Abstract

Forest biodiversity is essential for ecosystem health and provides critical services to humanity. However, threats from pollution and climate change underscore the urgent need for more accurate assessment methods. Monitoring and inventorying biodiversity are vital for informed decision-making in forest management, and Mixed Reality (MR) technology offers a promising solution to enhance traditional visual assessment methods. By overlaying 3D virtual information, such as text and holograms, in forest environments, MR can improve the contextualisation of biodiversity data. Here, we developed HoloFlora, the first interactive MR application designed to visualise biodiversity indicators on digital tree stems. With a geometric accuracy of 1.4 cm, HoloFlora sets a benchmark for future methods, demonstrating MR’s potential to deliver precise spatial information in complex forest settings. Expert evaluations validated the application’s intuitive design and functionality, and further refinements were made based on their feedback. The strong acceptance of MR technology among experts highlights its transformative potential in forestry, suggesting it could facilitate integration into existing management practices. Our findings establish a foundation for using MR in forest environments to enhance biodiversity monitoring and raise public awareness about biodiversity loss.

## Introduction

Mixed Reality (MR) is an emerging technology that is progressively transforming practices across various sectors, from industrial applications to leisure activities. MR is a medium that enables the seamless integration of virtual elements into the physical world. Its ability to visualise and interact with 3D virtual content has already revolutionised numerous fields. In education, for instance, MR facilitates the understanding of abstract concepts-such as the presence of molecular pollutants in air^1^ or the solar system organisation^2^-providing students with a more intuitive and engaging learning experience. Furthermore, MR has enhanced training methodologies by incorporating a “learning by doing” approach. Rather than relying on traditional manuals, users can now employ MR headsets that offer step-by-step guidance with interactive 3D instructions. This helps users to overcome the challenges of learning complex procedures, such as those involved in maintaining large industrial machinery^3^ or in assisting in surgical operations^4^. Recent advances have also extended MR’s applications to outdoor environments, including its use in building inspection^5^, cultural tourism^6^, and surveying^7^. However, the integration of MR technology remains limited in particularly challenging environments, such as forest settings. The high variability in light conditions, the dynamic nature of vegetation, and the uniformity of tones and colours in forest scenes complicate the deployment of MR sensors and systems^8^. Consequently, no dedicated commercial MR solution is currently available for forestry.

**Fig. 1:**
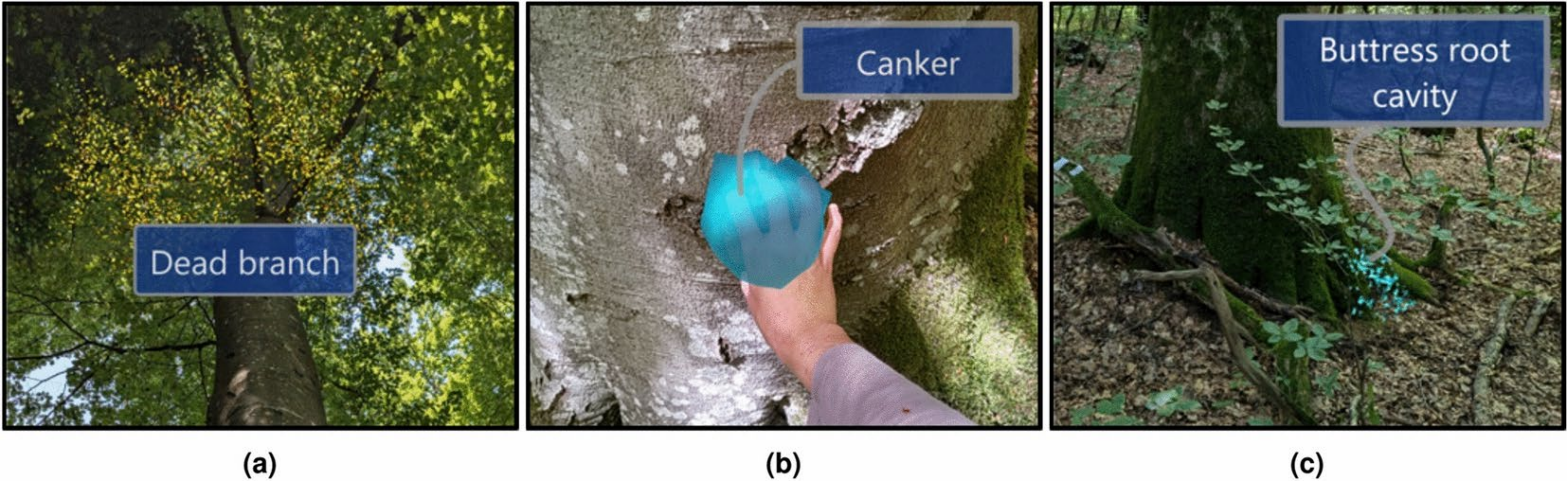
Mixed reality (MR) visualisation of biodiversity indicators on a single tree across forest layers: (**a**) canopy, (**b**) understorey, and (**c**) forest floor.

Despite these challenges, MR holds tremendous potential to transform forestry practices, by integrating remote sensing data from terrestrial or aerial sensors directly into forests, or by facilitating the extrapolation of detailed field measurements to larger areas under forestry management. This capability is particularly valuable in addressing the global biodiversity crisis. Forests, which harbour approximately 80% of the world’s terrestrial biodiversity^9^, are crucial to global conservation efforts. Further, temperate mixed forests in Europe are increasingly threatened by external pressures such as drought, storms and invasive species. In the face of climate change and ecological disruption, the ability to enhance forest resilience has never been more critical. The “close-to-nature” forest management strategy, which focuses on preserving biodiversity under these challenging conditions, has emerged as a promising approach^10^. However, it relies on accurate biodiversity assessments, which are currently based on traditional visual estimates, such as measuring deadwood profiles, counting habitat trees, and evaluating species diversity across forest plots^11^. While these methods provide valuable insights, they often lack spatial context and are prone to observer bias^12,13^. MR presents a more precise and immersive alternative by delivering enhanced spatial contextualisation (Fig. 1). This technology could significantly improve the efficiency and accuracy of biodiversity assessments, similar to its impact in other sectors^7^, by providing standardised, intuitive tools for education, training, decision-making and entertainment.

Recently, a methodology based on the MR headset sensors of the Hololens 2 was proposed to scan and estimate understorey vegetation structure^14^. While it lacks the precision to detect saplings and branches accurately, the proposed approach effectively identifies trees and has even been employed in a subsequent study to estimate habitat structures at camera trap locations^15^. This development underscores the growing interest in MR within the forestry sector, which has historically embraced technological advancements to enhance the efficiency and accuracy of forest management. A notable example of this trend is the digitisation of national forest inventories, with the aim to replace labour-intensive manual tasks with streamlined digital solutions^16,17^. Additionally, navigation in forest environments is becoming an important focus in robotics, with quadrupedal robots scanning the terrain^18^ and drones conducting environmental DNA (eDNA) measurements within the canopy^19^. In the long term, these advancements could greatly benefit from improvements in the spatial mapping and tracking capabilities of MR headsets.

To explore this potential, we developed HoloFlora, the first MR interactive application designed to visualise biodiversity indicators on digital 3D tree stems. The objective of this MR application is not only to enhance data visualisation but also to improve the process of learning methods of biodiversity inspection and evaluation in a more intuitive manner. To achieve this objective, we evaluated our solution from three key perspectives:

- Geometric accuracy: Assessing how accurately virtual elements align with the physical tree stems, and evaluating the stability of the tracking algorithm in forest conditions.
- User interface (UI): Evaluating the effectiveness and usability of the interactive UI and holographic elements through heuristic testing, thereby ensuring an intuitive and user-friendly experience.
- Technology acceptance: Gathering qualitative feedback from forestry professionals to assess the feasibility, practicality and potential acceptance of MR technology in forest environments, while discussing its broader implications for the field.

Through these evaluations, our work lays the groundwork for integrating MR into forestry for biodiversity conservation, while identifying additional applications that could impact forestry practices. Ultimately, our goal is to explore how MR technology can support forestry tasks, not only in learning and training but also in enhancing communication among forestry professionals. For instance, MR could offer clearer on-site perspectives on various forest management strategies, create seamless connections between past inventory data and current forest conditions, or enable more engaging dissemination of biodiversity knowledge through virtual guided tours.

## Methods

### Study area

**Fig. 2:**
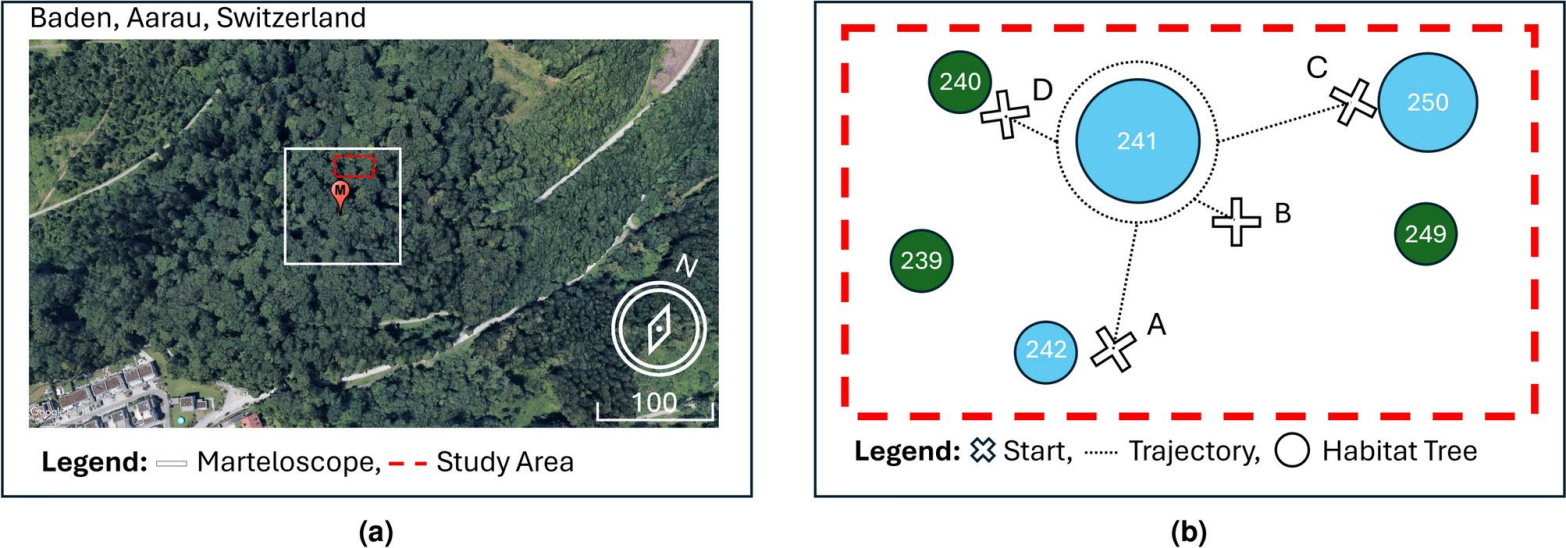
Top view of the study area: (**a**) satellite imagery of marteloscope (Google Earth Pro), (**b**) schematic representation with numbered habitat trees. Blue circles indicate common beech (*Fagus sylvatica*) and green circles represent silver fir (*Abies alba*).

Marteloscopes, derived from the French word *martelage* (tree marking), are instrumental in advancing MR integration into forestry. A marteloscope is a designated forest plot, typically spanning over 1 hectare, designed for training and learning purposes^20^. We conducted this study in the recently established Baden marteloscope, located in the Swiss canton of Aargau (Fig. 2a). This marteloscope is unique for its focus on tree-related microhabitat (TreM) identification exercises. TreMs are specific features of trees that provide essential ecological functions, such as providing shelter for insects, bats and birds or serving as water sources for mammals^21^. For our pilot study, we selected a 10 $\times$ 10 m area containing one habitat tree, i.e. a tree with at least one TreM, based on the feasibility of access around the stem (Fig. 2b). In addition, we prioritised the selection of a tree with a balanced distribution of TreMs from its base to its canopy. The selected tree featured two buttress root cavities at its base, a canker in the middle section, and a witch’s broom and some dead branches in the upper canopy.

### HoloFlora system

**Fig. 3:**
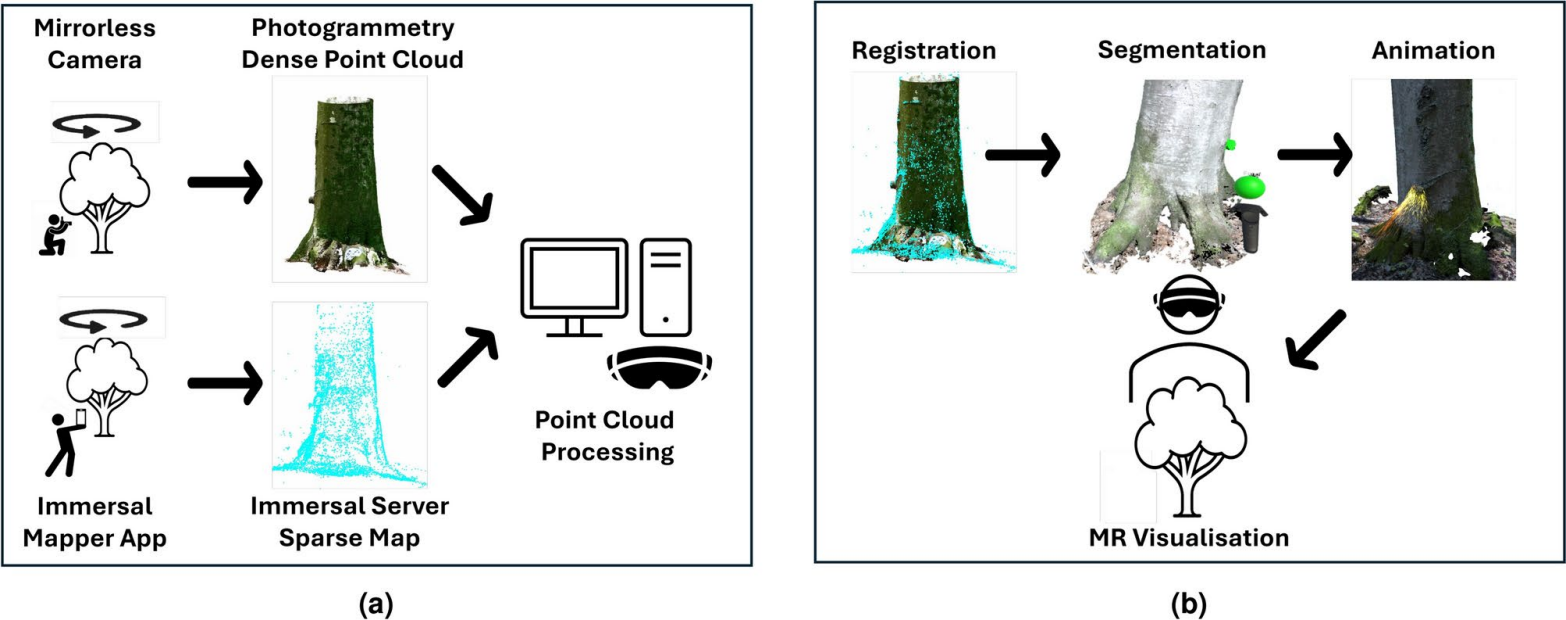
HoloFlora system overview, illustrating the workflow for: (**a**) tracking area setup and (**b**) digital content creation.

Figure 3 illustrates the methodology developed for integrating MR into forestry practices. The process is divided into two main phases: (1) tracking area setup (3a) and (2) creation of the digital content (3b).

1. Tracking area setup: To establish the tracking area, we employed the solution offered by Immersal (Immersal, Helsinki, Finland). We began the process by mapping the scene using the Immersal Mapper mobile app. We used an iPhone 15 Pro (Apple, Cupertino, CA, United States) in manual mode to capture images at approximately 1-meter intervals, consistently oriented towards the centre of the MR area. This approach, adapted from photogrammetry techniques, allowed us to map a 10$\times$10 meter area that included three trees. We sent the captured images to Immersal’s server for 3D map reconstruction. The Immersal map combined 2D features from the images with 3D data from the sparse point cloud, enabling precise camera pose tracking within the MR environment.
2. Digital content creation: For the MR experience, we drew inspiration from the Immersive Flora System^22^. Instead of targeting plants, we captured the tree stem using the same close-range photogrammetry (CRP) approach with a mirrorless camera (Nikon Z50, Tokyo, Japan). We processed the images in CloudCompare, where we manually aligned and refined the registration of the CRP sparse point cloud with the Immersal map downloaded from the server. Subsequently, we extracted biodiversity indicators, such as a cavity and a canker, using the VR labelling tool Labelling Flora^23^. Although we could have performed the extraction on a desktop computer, Labelling Flora proved to be faster and equally accurate. After we extracted ecological information from the trees, we georeferenced them during the Immersal map registration step. The final step involved using the Unity game engine (Unity Technologies, San Francisco, CA, United States). Specifically, we imported tree point clouds into Unity using the PCX package^24^ and animated them with artistic particle system visual effects. Additionally, we developed a UI using the MRTK2 toolkit, allowing users to toggle visual effects to minimise distraction from observing real biodiversity features. We then compiled and deployed the MR application to the see-through MR headset HoloLens 2 (Microsoft, Redmond, WA, United States).

### Geometric evaluation

#### Data collection

**Fig. 4:**
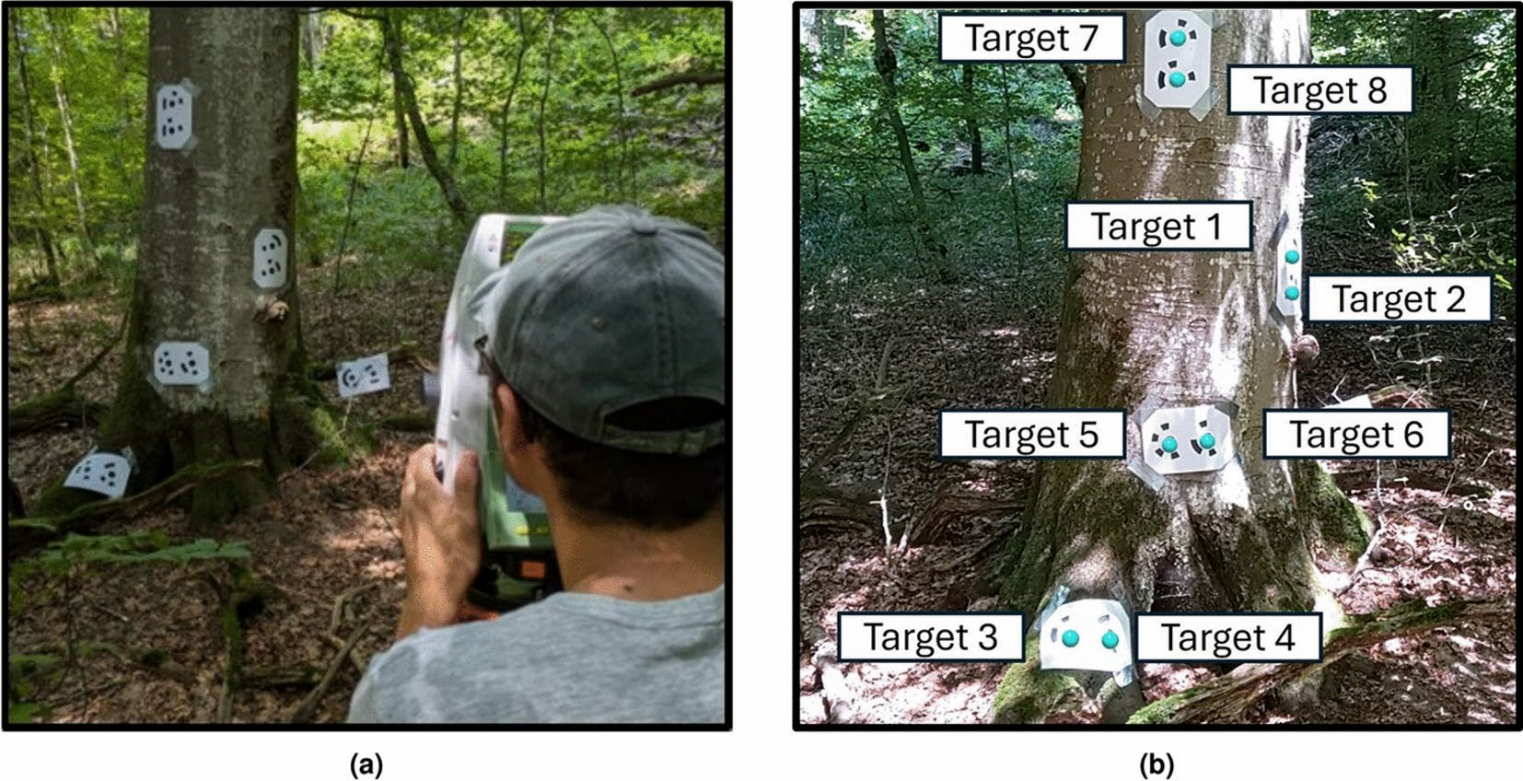
Data collection process. (**a**) Targets were placed on the tree stem and their positions were measured using a total station. (**b**) Holograms were aligned with the measured positions on the tree and captured using a mixed reality (MR) headset.

We conducted the data acquisition campaign for evaluating our MR forest methodology over two consecutive days. On the first day, as shown in Fig. 4a, we set up five pairs of coded targets (10 in total) on the habitat tree at the centre of the study area. Using a total station (TS06, Leica, Herrbrugg, Switzerland), we measured the distance to each target in a local coordinate system, achieving coordinate precision of $\pm 2$ mm. Simultaneously, we captured the tree and targets with close-range photogrammetry, using a mirrorless camera (Nikon Z50, Tokyo, Japan). We then reconstructed the 3D model of the tree in post-processing with RealityCapture (Epic Games Inc., Cary, NC, United States) while scaling it using the coordinates obtained from the total station measurements.

On the second day, we registered the high-accuracy 3D model with the sparse map created by Immersal. This registration allowed us to transform the photogrammetry point cloud into the Unity coordinate system. We did not perform absolute georeferencing, due to the poor quality of the Global Navigation Satellite System (GNSS) signal reception in the forest. We then manually extracted the centre position of each target from the georeferenced point cloud and displayed a holographic sphere in Unity at each centre. A C# script was initiated with the first successful localisation, continuously tracking the positions of the targets and recording them in a log file stored in the MR headset’s memory.

In the afternoon of the second day, we launched a version of the HoloFlora application containing only the spheres placed using our registration method, with no animations (Fig. 4b). This setup allowed us to track the position of each target. We conducted eight trials, each lasting 5 min. Figure 2b shows the starting point location in the forest plot and the trajectory we followed around the tree stem. We repeated each of the four paths twice.

We constrained the data acquisition to two days to ensure accuracy and consistency, i.e., to prevent target displacement and avoid long-term damage on the tree which would affect the analysis. However, one target shifted during the intermediary period, and we therefore excluded this pair of target acquisitions from the data logs. Additionally, we found that ambient light intensity affected the Immersal tracking algorithm, so we conducted the trials at the same time of day to maintain constant lighting conditions and thus achieve maximum accuracy.

#### Data analysis

We conducted two analyses to evaluate the accuracy and reliability of the virtual-to-physical world alignment:

- Assessment of the geometric accuracy and precision of the Immersal map.
- Evaluation of the stability and accuracy of the Immersal registration algorithm.

For the first analysis, we used CloudCompare (https://www.cloudcompare.org/, accessed 2024-08-22). The photogrammetry model scaled with the targets served as the ground truth. We employed the Multiscale Model to Model Cloud Comparison (M3C2) plugin to compute the accuracy and precision of the Immersal server map. Although the M3C2 plugin was initially developed for the analysis of complex topography^25^, it has also been proven effective for 3D tree stem point clouds^26,27^.

For the second analysis, we used a MATLAB (MathWorks Inc., Natick, MA, United States) script to analyse the log text files from the second day of data acquisition. Each log file contained 100 observations of all 9 target positions (at 3-second intervals over a period of 5 minutes). However, since tracking was lost a few times, the 100 measurements were not fully representative of the alignment. When tracking was lost, the spheres returned to their initial positions at the start of the application. To address this, we wrote a filtering function to remove the measurements of the target positions when they were not aligned with the real tree. This also allowed us to calculate the duration of target alignment during each trial.

### Expert evaluation

We configured HoloFlora to inspect a habitat tree from the Baden marteloscope. Before commencing the evaluation, we briefed the participants on MR technologies, the objective of the study, the potential risks associated with MR activities, and the voluntary and anonymous nature of the evaluation. We then asked them to complete and sign a consent form, and we collected demographic information including age and gender, as well as information about their prior experience with immersive technologies, to ensure transparency and ethical handling of participant data. The following subsections provide detailed descriptions of the user study.

#### Participants

The method and protocol for the expert evaluation received approval from the ETH Zurich Ethics Commission (registration number: EK-2024-N-88, approved on 18 May 2024). All methods were performed in accordance with the relevant guidelines and regulations. Before participating, all individuals provided informed consent after being fully briefed on the benefits and potential risks of each procedure. We recruited three participants, all experts in TreM identification, for a heuristic evaluation and a focus group discussion (FGD). While the sample size of three may seem small, it is well-suited for heuristic evaluations^28^. Additionally, the participants’ depth of experience and knowledge justified the smaller group size for the FGD^29,30^. The participants’ diverse backgrounds offered a broad range of perspectives on TreM identification. Although all participants were involved in teaching about TreMs, their professional experiences differed: Participant A worked in forest inventory for a canton, Participant B was a forest owner, and Participant C was a pioneer in TreM research. Their experience with MR also varied, as they rated their proficiency differently on a scale from 1 (Beginner) to 4 (Advanced): one participant rated themselves as 1 (Beginner), another as 2 (Intermediate), and the third as 3 (Good). This highly qualified group of TreM experts aligns with what is referred to in the literature as a “mini group”, which is considered a valid approach for generating insights from experts^31^.

#### Measures

To evaluate the integration of MR technology in forestry, we focused on two key measures: usability and technology acceptance. Usability assesses how well MR technology adapts to forest environments, while technology acceptance gauges whether foresters recognise tangible benefits from its use. Details of these measurements are outlined below. We completed the entire evaluation process within half a day.

*Usability*. We assessed HoloFlora’s usability through a heuristic evaluation, which has become a standard for evaluating prototypes and early versions of applications. Originally designed for general UI evaluation, this method has been adapted over time to accommodate various types of interfaces. For our study, we used the Derby Dozen AR/MR Usability Heuristic Checklist, a comprehensive toolkit specifically created to evaluate the usability of augmented reality (AR) and MR devices and applications^32^. The checklist includes 12 heuristics and 109 items. Since our focus was solely on an MR application rather than on the device itself, we adapted the checklist by excluding four heuristics (1. unboxing and setup; 2. instructions; 10. collaboration, and 11. privacy), as they were not relevant in our context. This adaptation left us with 72 items. We further refined this list by removing items marked as not applicable (N/A) to our application, resulting in a final set of 40 heuristic items for the evaluation, listed in Table 3.

*Technology acceptance*. To evaluate MR acceptance in forestry practice, we employed the Technology Acceptance Model (TAM)^33^. Figure 8 illustrates the TAM, where the behaviour (actual use) can be predicted based on the perceived ease of use (PEOU), perceived usefulness (PU) and behavioural intention (intention to use). TAM typically involves questionnaires to measure the PU and PEOU of the technology. While these questionnaires quantify user intentions for the technology, they often lack depth in explaining specific reasons for acceptance or rejection. Therefore, to gain a more comprehensive understanding of the motivations and challenges of integrating MR into forestry, we complemented the Likert-scale items with a qualitative approach by conducting an FGD with three forestry experts, using open-ended questions to gather detailed feedback. This combined method allowed us to categorise feedback according to TAM constructs, while also providing in-depth insights into the underlying reasons. This offered a thorough understanding of MR’s potential in forestry and a more nuanced assessment of user intentions.

#### Procedure

After the participants completed the briefing and signed the consent form, they were given a Hololens 2 MR headset (Microsoft, Redmond, WA, United States) and instructed to initiate the eye calibration process. Following this, participants engaged with the HoloLens Tips tutorial, designed to introduce them to the MR features of the system. The aim of this procedure was to enhance the immersive experience by optimising visual clarity, minimising the risk of cybersickness, and establishing a consistent baseline of interaction for all participants before the evaluation, which comprised two distinct phases: heuristic evaluation and FGD.

Each participant conducted the heuristic evaluation individually, immediately following their MR introduction. They reviewed all 40 items on the heuristic evaluation sheet provided. After confirming their understanding of the evaluation criteria, they launched the HoloFlora application from the Hololens menu and were guided through its functionalities for inspecting biodiversity on habitat trees.

Participants were required to complete specific tasks:

- Task 1: Open and close the hand menu.
- Task 2: Activate and deactivate the visual effects.

Following each task, participants verbally confirmed its successful completion. They were then free to explore the habitat tree and experiment with the visual effects. Upon completing the full inspection of the habitat tree, participants removed the headset and completed the questionnaire sheet. They were also given the option to restart the application if needed to resolve any uncertainties. The heuristic evaluation allowed us to gather detailed feedback on the HoloFlora application and to identify potential bugs and limitations.

We conducted the FGD on the same day as the heuristic evaluation, with the same three experts. The discussion began with a reminder of its objective: to gather feedback on the integration of MR in forestry. We informed the participants that their insights into both the advantages and limitations of MR in this context were equally valuable. At the end of the discussion, we invited the participants to provide suggestions for improving the HoloFlora prototype. Finally, we thanked the participants for their involvement and reminded them of the confidentiality of their responses.

## Results and discussion

In this section, we present the evaluation results and their implications for integrating MR into forestry practices. We begin with a technical analysis of HoloFlora, including real-virtual alignment validation through the M3C2 comparison and hologram stability analysis. Next, we evaluate the HoloFlora user interface, focusing on its usability and effectiveness. Insights from a FGD provide further context, helping to assess our hypotheses on the potential applications of HoloFlora in forestry. These findings demonstrate the feasibility of MR for biodiversity conservation and offer insights into its broader adoption in the field

### Geometry analysis

**Fig. 5:**
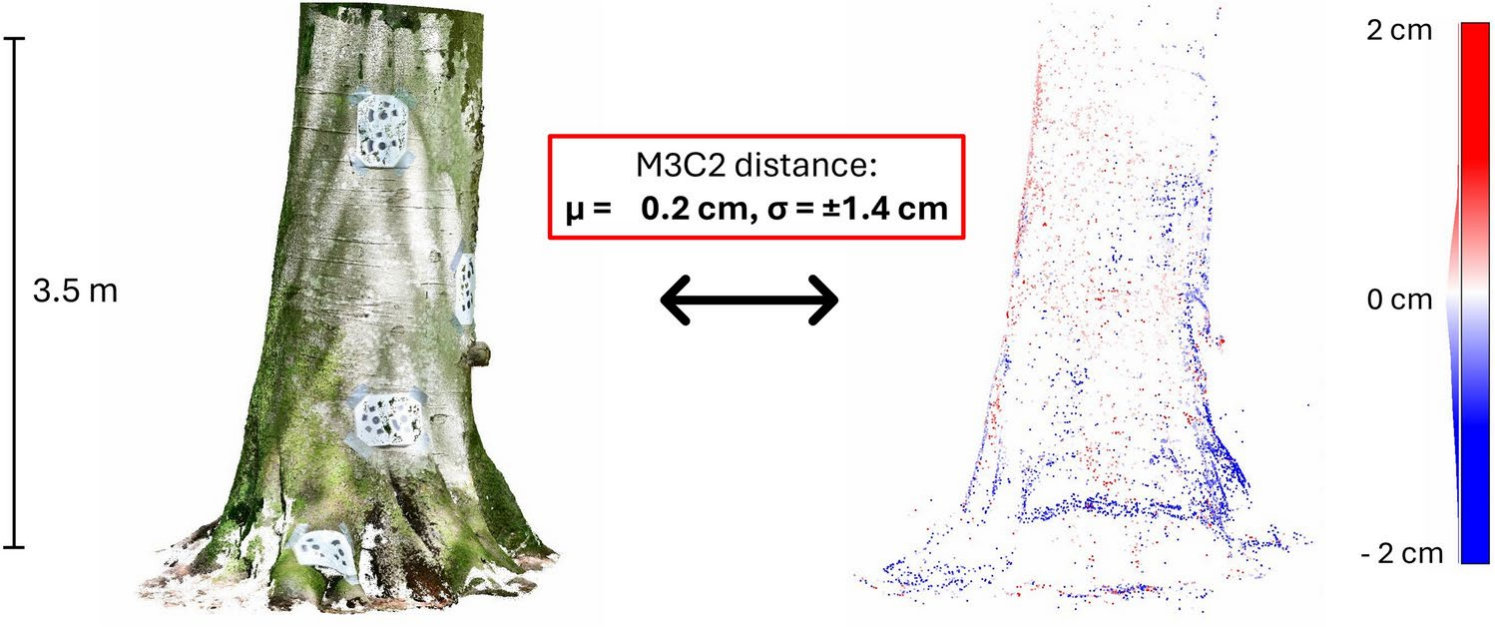
Multiscale model-to-model cloud comparison (M3C2) with a coloured dense photogrammetry point cloud as a reference (left), and a sparse Immersal map with blue, white and red colours derived from the M3C2 computation (right).

In Fig. 5, we observe that the sparse Immersal map, utilised for tracking the positions of the MR headset, aligns closely with real-world tree stems, exhibiting a maximum margin of error of $\pm 2$ cm. To gain a clearer understanding of this alignment, we calculated the mean distance ($\mu$) and standard deviation ($\sigma$) between the virtual elements and their real-world counterparts. The obtained mean distance of 0.2 cm indicates that our methodology for aligning the digital model with the actual tree is highly reliable, while the standard deviation of 1.4 cm confirms the precision of the 3D content alignment for biodiversity assessments.

To put these results into perspective, in the case of the smallest TreM, specifically the canker, which fits within a box measuring 10 cm $\times$ 10 cm $\times$ 10 cm, a deviation of 1.4 cm is more than reasonable for accurately locating it on the tree stem. Therefore, these findings validate the methodology employed by the HoloFlora system for accurately visualising TreMs. However, it is important to note that drift can commonly occur after the application starts, which may affect the alignment of the virtual elements with the real world. Thus, it is essential to consider the stability of the hologram position. The subsequent analysis will provide further insights into this issue.

Figure 4b in the “Methods” section illustrates the habitat tree overlaid with holograms at various heights. We positioned the targets to adequately cover the tree and represent the variations in TreM location in biodiversity inspections. Table 1 shows the stability of the holograms along the three axes, with mean values of X = 0.5 cm, Y = 0.5 cm, and Z = 1 cm. However, during the recording the tracking was occasionally lost for a few seconds due to changes in lighting on the tree stems. This is also evident in Fig. 4b, where a white hollow appears on the tree stem. When tracking was lost, the positions of the spheres were not recorded. Nonetheless, we observed that the algorithm was able to reset itself after moving around the tree.

These results confirm the reliability of HoloFlora’s spatial alignment for TreM identification at the single-tree level. However, to enable broader area coverage, Immersal also supports the integration of laser scanning point clouds as spatial maps. While photogrammetric point clouds were preferred in this study for their ability to capture TreM’s fine-scale details, future research could explore the use of laser scanning data to scale up the approach for biodiversity assessments at the plot or forest level.

**Table 1:** Summary of geometric error on targets over a recording lasting 5 min.

Target standard deviation (cm)									
#	1	2	3	4	5	6	7	8	Mean
X	0.5	0.5	0.6	0.6	0.4	0.4	0.7	0.6	0.5
Y	0.4	0.4	0.7	0.7	0.6	0.5	0.5	0.5	0.5
Z	0.7	0.8	1.1	1.1	0.9	0.9	1.2	1.1	1.0

### HoloFlora analysis

**Fig. 6:**
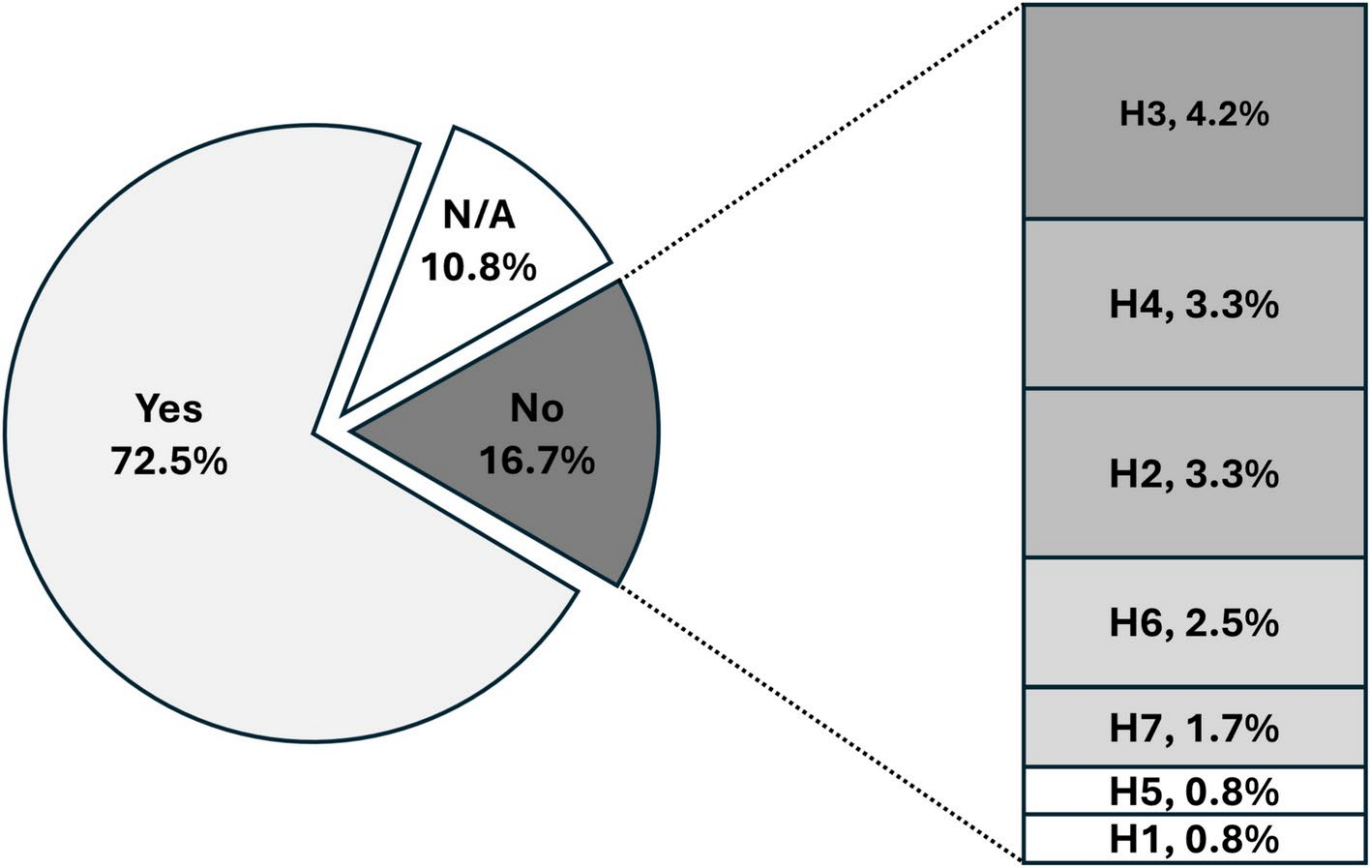
Heuristic usability questionnaire results from three expert participants. The pie chart illustrates the proportion of ‘Yes’, ‘No’, and ‘Not applicable (N/A)’ responses, and the bar plot presents the distribution of ‘No’ responses across seven heuristic criteria: H1 (organisation and simplification), H2 (consistency and flexibility), H3 (integration of physical and virtual worlds), H4 (user interaction), H5 (comfort), H6 (feedback to the user), and H7 (intuitiveness of virtual elements).

**Fig. 7:**
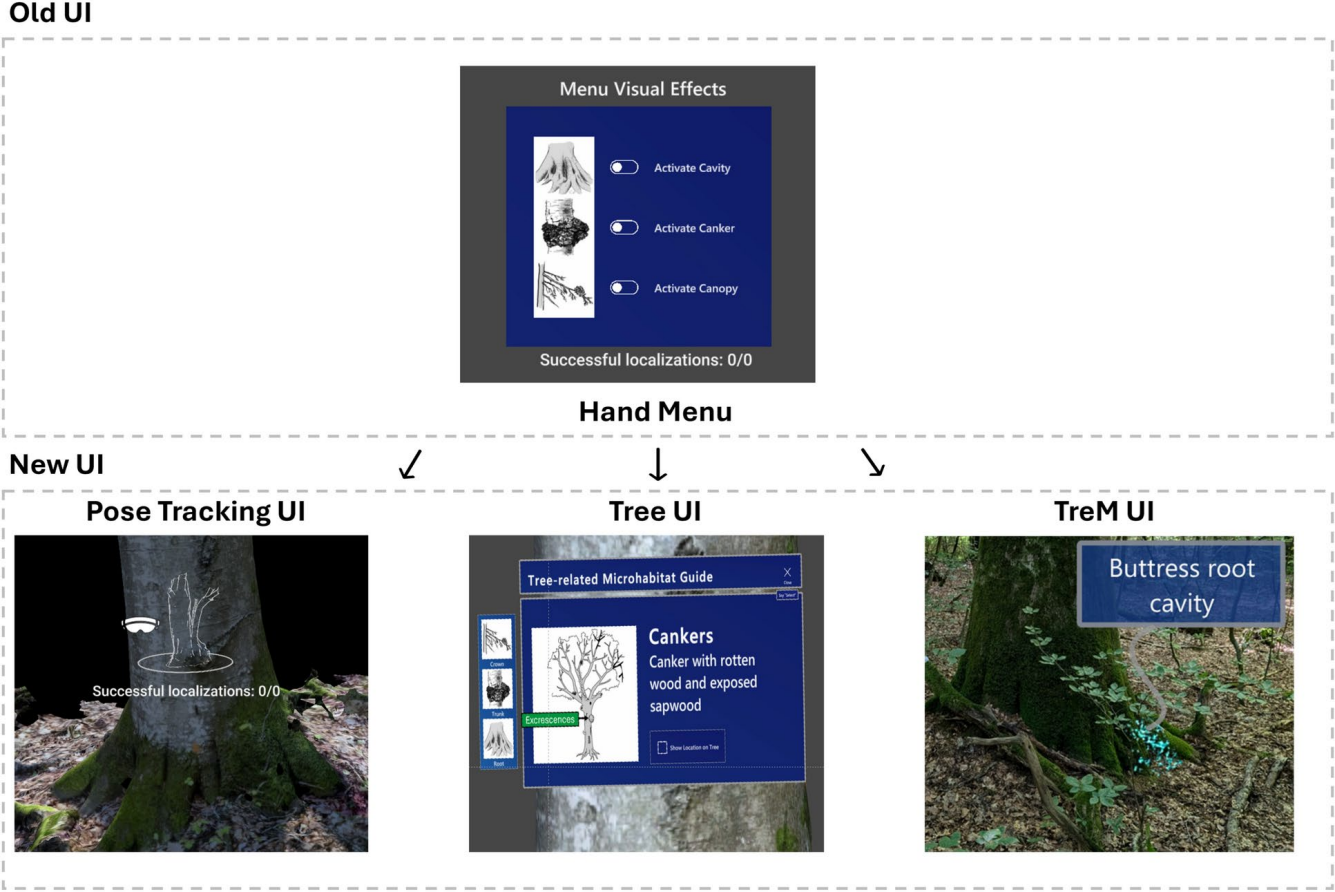
HoloFlora user interface (UI) design modifications based on expert feedback.

The pie chart in Fig. 6 gives a summary of the heuristic questionnaire responses from the three forestry experts. The large proportion of affirmative responses indicates that HoloFlora was generally perceived as well-implemented and user-friendly in terms of usability. The small proportion of N/A responses suggests that most of the questionnaire items were considered valid for the evaluation, although some refinement may be necessary. The negative responses are particularly informative, as they highlight areas of the prototype that require improvement, aligning with the primary objective of the heuristic evaluation to identify and address usability issues. The bar plot categorises the negative responses according to the corresponding heuristic categories. The data suggests that the most effort should be focused on improving the ‘integration of physical and virtual worlds’ (H3), with five negative responses. The next priorities are ‘consistency and flexibility’ (H2) and ‘user interaction’ (H4), each with four negative responses, followed by ‘feedback to the user’ (H6), with three negative responses. Addressing these areas could potentially resolve 80% of the negative feedback. For this, we considered the comments from the users and summarised them into three potential improvements:

- Tracking system: a notable challenge encountered with HoloFlora was the recurrent loss of tracking. When tracking is lost, the system resets the virtual content to its initial position, prior to alignment with the real tree. This misalignment can confuse users, as they may perceive the hand menu buttons as non-functional, when in fact the content is displayed outside their field of view. As shown in Fig. 7, we propose mitigating this issue by disabling the hand menu temporarily and displaying an alert animation, prompting the user to re-establish tracking. A more advanced approach could involve the implementation of a filtering algorithm, such as a Kalman filter. Commonly used in navigation systems, this technique could help to minimise tracking drift over time, thereby enhancing the stability of the virtual content and reducing alignment errors.
- UI: Another notable concern raised by the experts was the obstructive nature of the hand menu, which blocked the user’s view while moving, as well as the difficulty in deactivating it. Beyond the menu’s positioning, there were additional comments on the aesthetics and content, particularly the need for clearer text and button descriptions and for better image quality. To address these concerns, we developed a second version of the hand menu, as shown in Fig. 7. This updated menu should improve usability by providing more detailed information and refining the visual presentation, thereby enhancing the overall user experience.
- TreM 3D graphics: Feedback on the TreM 3D graphics highlighted the particle systems as engaging, yet there were suggestions for further enhancement. The experts recommended adding more 3D graphics content and providing clearer information about the relationship between the virtual and real versions of TreM. In response, we added a tooltip UI displaying the name of the TreM above the virtual effects, as depicted in Fig. 7. We also plan to incorporate sound effects corresponding to the TreMs. These sounds will play upon activation and deactivation, providing auditory feedback to users. Additionally, incorporating sound could enhance the immersive experience in HoloFlora. For instance, replacing floating particles in the canopy with bird sounds could provide a more coherent and realistic virtual representation.

### Technology acceptance

Through our FGD, we gathered valuable insights from the participants regarding both the limitations and applications of MR in forestry practices. We organised the experts’ opinions in Tables 2 and 4, and we discuss them in the following subsection.

**Table 2:** Major limitations raised during the focus group discussion (FGD).

Limitation	Description of the limitation	Supporting literature
Technical:	**Headset Ergony:** *“if you look at the side, I see the reflection of* *what’s around you in the lenses”.* (Participant A)	Microsoft 2022^34^
	**Field of view:** *“on very tall trees where some microhabitat* *might be a bit on the higher part of the tree, it’s difficult to spot”.* (Participant B)	Hockett and Ingleby 2016^35^
External:	**Weather condition:** *“That would be a question. If you get* *raindrops on the lens, does it matter”.* (Participant C)	Microsoft 2024^36^
	**Relationship to Nature:** *“society is getting further away from* *anything that is on the production side.(...) but also in forestry* * or in forest timber production. (...) if it’s getting too far away (...)* * then people lose the feeling of what does it mean to chop a tree (-)”.* (Participant C)	Hu and Fol 2024^22^

Table 2 summarises the major limitations identified by the experts. Although they were generally satisfied with the device’s weight and portability, a notable drawback was attributed to the lenses. In forest environments, the HoloLens 2 was reported to be susceptible to sunlight reflections, which can impair visibility considerably and thereby diminish the technology’s overall effectiveness. Additionally, the field of view was perceived as too restrictive, particularly when assessing larger sections of trees, such as the canopy. While these limitations are acknowledged in the manufacturer’s technical documentation, the experts’ feedback provides valuable insights into specific headset features that could be potential obstacles in forestry applications.

Another significant concern involved the adaptability of MR technology to diverse weather conditions particularly in forests with more extreme climates than the alpine forest tested, such as boreal, tropical, or cloudy forests. Experts expressed scepticism about the reliability and performance of the device under adverse weather conditions, particularly rain, which could disrupt both tracking and display functions. While using smartphones or tablets with waterproof cases could be a solution, it was noted that similar challenges would persist. Rain could obscure the screen and interfere with touchpad functionality, while fog could limit the field of view, even if the system itself functions properly. This suggests that resolving these issues may simply require sufficient customer demand in the forestry sector for manufacturers to adapt current solutions, as seen with the Trimble MR helmet for construction and HoloLens 2 Industrial Edition for clean room or hazarduous location.

The FGD also explored the broader implications of integrating MR technology into natural environments. One participant raised the concern that such integration could alter the user’s connection to nature. For instance, conducting virtual tree-cutting exercises might reduce the emotional significance of such actions. Nonetheless, it could be argued that MR offers an optimal balance between technological advancement and the preservation of natural experiences. Holoflora, for example, is designed to enhance biodiversity by revealing TreMs through artistic particle effects that are otherwise invisible elements of the forest environment. In this context, MR technology has the potential to deepen the human–nature connection. Moreover, by incorporating sound, the immersive experience could be further enriched, fostering a more profound understanding of biodiversity among forestry practitioners.

Despite these identified limitations, it is encouraging that the experts unanimously agreed on the future potential of MR technology in forestry. None of the experts struggled to use HoloFlora, including the two without a technical background. They expressed confidence that the current challenges are likely to be addressed in future iterations of the technology and should not impede its adoption. Overall, the experts were highly receptive to the potential of MR. They provided several valuable examples of applications, which are elaborated upon in the following subsection.

Table 4 presents the most promising applications of MR technology in forestry, as identified through FGD. Using the TAM criteria developed by Davis et al. (1989), we systematically categorised the experts’ feedback. Only those applications meeting both PEOU and PU criteria were included in the table. These applications -in training, learning, entertainment and decision-making- demonstrate the broad applicability of MR. Notably, MR was envisioned for diverse uses, including decision-making support for forest stakeholders, interactive training guides for practitioners, and audioguides for hikers and forest visitors. Additionally, responses to the FGD question “All things considered, why would you recommend or not recommend using MR for facilitating the learning of TreM identification?” emphasise the value of MR in education (see Annex).

This segment of the FGD underscores a consensus among the experts regarding the huge potential of MR technology, despite certain technical and logistical challenges. MR technology shows considerable promise for advancing forestry education, with one university lecturer (Participant C) already expressing a clear intention to integrate it into their curriculum. This highlights the added value of MR in facilitating the dissemination of ecological insights within forest management practices. However, it is important to emphasise that MR should not replace traditional field trips and in-person visits to forests. MR can serve as a valuable complement to such excursions by enhancing the natural environment with visualisations of otherwise invisible elements, such as biodiversity, below-ground water, or CO_2_ sinks.

## Conclusions

The presented study establishes a strong foundation for the integration of MR into forestry practices, demonstrating the technology’s potential to transform how biodiversity is inspected on tree stems and how forestry operations are managed. A precise and stable methodology was developed and validated through expert feedback, achieving a virtual-to-physical world coordinate transformation accuracy of up to 1.4 cm. It can therefore be considered suitable for a wide range of applications, from training and education to entertainment and decision-making, as highlighted by forestry experts. However, a notable limitation was the tracking instability caused by fluctuating light conditions, particularly under sparse canopy cover. Additionally, the stability of hologram placement over a 5-minute trial was very promising ($\sigma = 1.4$ cm). However, we observed the need for improvements in relocating holograms when tracking is lost and in optimised the UI to notify users when tracking errors occur.

These observations led to the development of an optimised HoloFlora prototype for TreM identification training at the single-tree level. The HoloFlora methodology is highly generalisable, using 3D point clouds as input and deployed on the platform-agnostic Unity engine. Beyond TreM identification training, MR has broad potential applications in forestry, as highlighted by experts:

- **Education**: a MR application for visualising past forest inventory data could reduce observer bias and improve understanding of forest evolution. Images or point clouds from prior inventories could be deployed on MR headsets or affordable tablets.
- **Entertainment**: an MR application providing an interactive forest guide could raise public awareness about biodiversity loss. Large point clouds obtained through mobile laser scanning could be combined with smartphones, ensuring the immersive experience is accessible to a broader audience of visitors.
- **Decision-making**: an MR application for accessing a digital twin of the forest could facilitate collaboration between office-based users and on-site practitioners. Office users could use XR video see-through headsets with a wider field of view to interact with terrestrial and aerial remote sensing data, and guide on-site users’ MR headsets to recognise or record observations of target features.

Ultimately, MR could play a vital role in reconnecting people with nature by improving their ability to recognise, understand, and appreciate the processes and ecosystems within forests, even those hidden to the naked eye. This deeper understanding could foster stronger public support for sustainable forest management and broader efforts to combat biodiversity loss, making MR as a valuable tool in environmental education and conservation efforts.

## Supplementary Information

Supplementary Information.

## Data Availability

All data used in this study can be made available upon request to the corresponding author. The HoloFlora application is accessible from a GitHub repository, accessible through this DOI: https://doi.org/10.5281/zenodo.14025501, hosted on Zenodo.
