# Supplementary material for: Advancing forest biodiversity visualisation through mixed reality
Source: Sci Rep. 2025 May 7;15:15908. doi: 10.1038/s41598-025-00285-y (PMC12059045; doi:10.1038/s41598-025-00285-y)
Supplement: Supplementary file 1 — Supplementary Information. [file 41598_2025_285_MOESM1_ESM.pdf]

Additionnal information

Figure

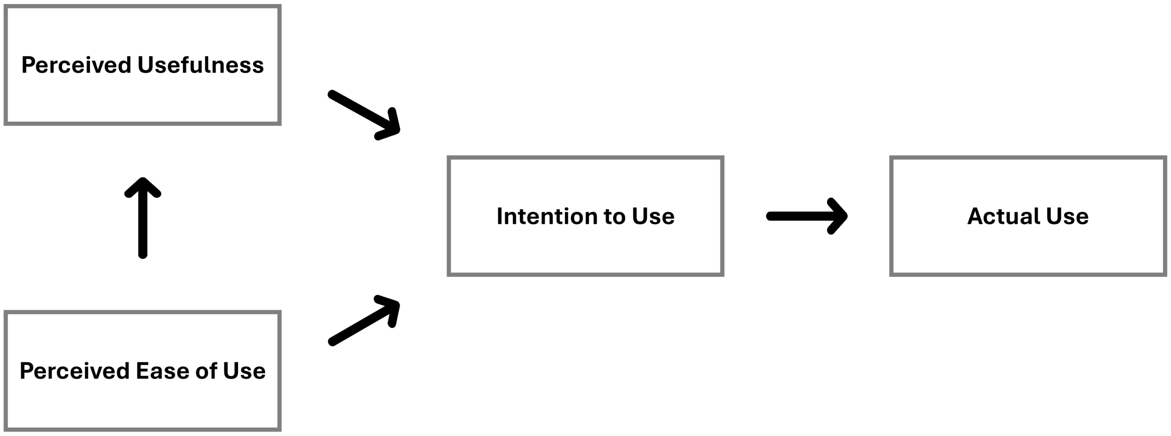

Figure 8. Technology Acceptance Model (TAM) (Davis, 1989[33]).

Table

Table 3. Summary of heuristics for enhancing user experience in applications.

Heuristic	Definition	Items
Organisation & Simplification	The application should minimise cognitive overload by easing the user into the environment and avoiding unnecessary clutter.	6
Consistency & Flexibility	The application should be consistent and follow design standards for text, audio, navigation and other elements.	7
Integration of Physical & Virtual Worlds	It should be easy to identify virtual elements and to determine which virtual elements are intractable. Virtual elements should not obstruct physical objects in the users' environment that are crucial for the completion of their goals.	7
User Interaction	All interactions that the user has with application should be simple, easy to understand, and easy to complete.	8
Comfort	The application should provide adequate feedback to the user to explain what is currently going on.	5
Feedback to the User	The application should provide timely and clear feedback to inform users of their actions and system status.	3
Intuitiveness of Virtual Elements	The application should be designed in a way that promotes the use of recognition rather than recall, to minimise the user's memory load.	5

Table 4. Future perspectives for MR development in forestry based on expert feedback.

Application	Perspective
**Training:**	• **Perceived Ease of Use:** *“(-) in some cases, where you are not completely sure if one microhabitat is rather falling into one category or another, and having this kind of description [virtual information] might also help even practitioners in that case. To better understand how a microhabitat looks like, which characteristics does it need to have, to fall under specific categories, for example”.* (Participant B)<br>• **Perceived Usefulness:** *“(-) a great tool for training people that are not normally used to seing some certain types of microhabitats (-)”.* (Participant B)
**Learning:**	• **Perceived Ease of Use:** *“Having the hands free is actually very practical because, [...], you need your hands to move around tree, and being able to visualise the things without using your hands or having the tablet in your hands is a very good thing, actually”.* (Participant B)<br>• **Perceived Usefulness:** *“(-) it can be really useful [not only] for people that work in the forest, [...] but also for people which are not familiar with the forest and just go occasionally for a hike or so. [...] like a guided tour, where you could get some additional information [...and] explain the importance of forests to a broader community”.* (Participant A)
**Entertainment:**	• **Perceived Ease of Use:** *“And also it kind of approaches also young people that might be, let’s say, closer to new technologies (-)”.* (Participant C)<br>• **Perceived Usefulness:** *“(-) [Mixed reality] can be for sure a good way of [...] taking people and [... their] curiosity and use it for the greater good and bringing people into the forest to learn more about microhabitats (-)”.* (Participant B)
**Decision-making:**	• **Perceived Ease of Use:** *“(-) Mixed reality has potential and it should be further developed [...] one of the real potential I would see is to connect different ecosystem services to nature conservation [...] if you think about tree cavities or mould cavities that store moisture and maybe they are even attached to the ground. Then [...] these are important structures for the forest climate, but also for the water storage in the forest. And that could be brought together probably at a certain point”.* (Participant C)<br>• **Perceived Usefulness:** *“one could really also discuss more social [aspects]. And, let’s say, decision making, which is usually crucial in forest management. Because you may ask ten foresters and you get ten different solutions, but probably none of them is wrong in a way.”* (Participant C)

Interview extract

- "(—) Yeah, I think it's definitely something that has a potential and it should be further developed. Absolutely! And one of the real big potentials, I would say, is also to connect the different ecosystem services to nature conservation aspects. (—) To connect them [tree-related microhabitats] also with (—) the CO2 sink. Also water, (—) if you think about tree cavities or mould cavities that store moisture, and maybe they are even attached to the ground. These are important structures for the forest climate, but also for the water storage in the forest. (—)" (Participant C)
- "Yeah. I also think it [has potential,] especially into the teaching education part. [It] doesn't necessarily have to be academic, but also for the general public. Given also the interest that you have more and more into mixed reality aspect. (—) [MR] can be for sure a good way of (—) taking curiosity and use it for the greater good and bringing people into the forest and learn more about microhabitats. (—) Or practitioner[s] (—) can increase their knowledge on microhabitats and how to deal also with decision[s] related to microhabitats." (Participant B)
- "Yeah I agree, especially for marteloscopes, (—) [or] for teaching practitioners, (—) students [or] (—) higher positions. Yeah, there is great potential, but [...] it needs some development [...] this will be a matter of time." (Participant C)
- "And could you imagine yourself using it for the future development of your lectures?" (Moderator)
- "Yeah, definitely! Yeah, it would be very cool, if we could also offer this here for the students. I mean, it's a logistics question. You need a lot of goggles and so on. But if it would be possible, I think it would be very interesting to do a full day excursion. (—)" (Participant A)

Competing interests

The authors declare no competing interests.
